# The Influence of Women’s Empowerment on Poverty Reduction in the Rural Areas of Bangladesh: Focus on Health, Education and Living Standard

**DOI:** 10.3390/ijerph18136909

**Published:** 2021-06-27

**Authors:** Wei Wei, Tanwne Sarker, Wioletta Żukiewicz-Sobczak, Rana Roy, G. M. Monirul Alam, Md. Ghulam Rabbany, Mohammad Shakhawat Hossain, Noshaba Aziz

**Affiliations:** 1 School of Economics and Finance, Xi’an Jiaotong University, Xi’an 710049, China; wei_wei@xjtu.edu.cn; 2 Department of Food and Nutrition, Calisia University, 62-800 Kalisz, Poland; 3 Department of Agroforestry & Environmental Science, Sylhet Agricultural University, Sylhet 3100, Bangladesh; ranaroy.aes@sau.ac.bd; 4 Department of Agribusiness, Bangabandhu Sheikh Mujibur Rahman Agricultural University, Gazipur 1706, Bangladesh; gmmonirul79@gmail.com; 5 College of Economics and Management, Northwest A&F University, Yangling 712100, China; tonoy_mkt@nwafu.edu.cn (M.G.R.); shossain7490@gmail.com (M.S.H.); 6 Department of Agribusiness and Marketing, Sher-e-Bangla Agricultural University, Dhaka 1207, Bangladesh; 7 Economic Development and Value Chain Specialist, World Vision Bangladesh, BleNGS Project, Jamalpur 2000, Bangladesh; 8 College of Economics and Management, Nanjing Agricultural University, Nanjing 210095, China; noshabaaziz@yahoo.com

**Keywords:** Bangladesh, gender violence, multidimensional poverty, poverty reduction, women’s empowerment

## Abstract

Women’s empowerment has a great influence on health, nutrition, education, and the overall well-being of societies as well as of the children and households. This study investigates the effect of women’s empowerment on poverty reduction and focuses on household deprivation, in terms of education, health, and standard of living. Primary data was collected from 914 married women from rural areas of Bangladesh using a well-structured questionnaire and a random sampling technique. Descriptive statistics, logistic regression, and ordinary least squares models were used in this study. The results indicate that increased women’s access to education, asset ownership, decision-making power on children’s health and education, and access to medical facilities, have caused a significant decline in income poverty and multidimensional poverty. However, gender violence, taking resources against women’s will, and preventing women from working outside, have caused a considerable decline in per capita income and an increase in income poverty and multidimensional poverty. Overall, it is found that women’s empowerment has a great impact on the reduction of income poverty and multidimensional poverty in society. The findings of the study can assist and guide policymakers to initiate appropriate strategies for women’s empowerment to reducing poverty in Bangladesh while making progress towards other social and developmental goals.

## 1. Introduction

Achieving gender equality is regarded as one of the key goals of the 2030 Agenda for Sustainable Development, as women and girls continue to suffer serious discrimination problems in every part of the world [1]. The vital aspect of the goal of women’s empowerment is connected to improving health and nutrition status, ensuring food security, eliminating hunger, and reducing poverty [2,3]. “Poverty is a pronounced deprivation of well-being”, whereas well-being is measured by education, assets, housing, health, nutrition, and certain human rights in society [4]. Empowering women and enhancing women’s status can play a significant role in the achievement of many development programs and help to bring positive societal transformation [5]. Globally, women constitute about 43% of the total agricultural labor force [6] and also contribute to 50% of the total food production [7]. It has been found that when more power is given to women to use household income, the proportion of money spent on healthy food increases because women favor spending more money on nutritive and high-quality foods than on unhealthy foods, recreation, and alcohol [8,9]. Women’s empowerment is frequently cited as a goal of rural development aimed primarily at reducing household vulnerability to poverty and food insecurity [10,11]. Empowering women is also a vital instrument in the fight against poverty [12,13].

Bangladesh has been struggling to reduce the prevalence of poverty and to improve the socio-economic conditions of poor citizens. In the early 1970s, the percentage of people living below the poverty line was 80%, and in 2016, this percentage decreased by 24.3%. Moreover, the employment and literacy rates of women have increased from 36% and 57.8% in 2011 to 88.5% and 69.5% in 2016, respectively [14]. The changes and transformations in women’s lives are broadly seen as the cause and consequence of considerable human development over the past 25 years in Bangladesh [15]. Although women constitute about half of the Bangladeshi population, their social status, especially in rural areas, remains very low. Rural women are among the most disadvantaged members of society, suffering from social oppression and economic inequality; the vast majority of them are impoverished [16], and their empowerment, therefore, is critical to bringing about a positive change in their lives.

A great number of empirical studies have investigated the impact of women’s empowerment on education, child health, food security, and nutrition [17,18,19,20]. However, our study specifically evaluated the impact of women’s empowerment on household income poverty and multidimensional poverty, by focusing on the rural areas of Bangladesh. This study extends previous work by providing new empirical evidence on the impact of women’s empowerment on their overall livelihood status. This study is a contribution not solely to descriptive literature on the present condition of women in Bangladesh, but also offers critical insights into the effects of women’s empowerment on their households.

## 2. Background of the Study

Considerable diversity exists in the foci, agenda, and terminologies used to define women’s empowerment [21]. The terms, “women’s empowerment”, “gender equality”, “female autonomy”, or “women’s status”, are widely used in literature, and all these terms focus on women’s power and control in making life choices [21]. Women’s empowerment is the process whereby women learn knowledge and skills, overcome difficulties, and benefit from useful resources [22]. It is not only an outcome but also an intermediate variable to further observe other developmental outcomes, especially poverty [23]. The idea of empowerment is often linked to the notion of power relations, which specifically refers to gaining either more or less power among actors [24,25]. However, women’s empowerment is different from the empowerment of other disadvantaged groups due to intra-household dynamics [26,27]. Women’s empowerment and gender equality are often considered two sides of the same coin: progress toward gender equality requires women’s empowerment, on the other hand, women’s empowerment contributes to increasing gender equality. In the case of gender inequality or discrimination, it is generally believed that women are excluded or disadvantaged in terms of decision-making and access to economic and social resources [28]. All definitions of women’s empowerment make some reference to women’s ability in certain areas, specifically in controlling their own lives, having the freedom to make decisions, and having the input to change life choices [27]. Women’s empowerment is typically conflated with the status that women have, which is often represented by the acquisition of and control over resources [29], which not only include material and financial resources, but also the social and human resources that can increase a woman’s ability to exercise her choices [30]. Examples of such resources include age, education, employment, social capital, networking, and ownership of property. Sharaunga et al. [31] reported that empowerment is multidimensional, and women empowered in one dimension do not necessarily empower in another. It has also been established that women require both resources and a sense of agency to achieve their live outcomes on their own. The multidimensional concept of the agency includes different domains, namely, decision-making authority, control over finances, and freedom of movement. Having the authority over making decisions and overusing money, is a more apt and precise description of women’s power. Although there is no single definition of women’s empowerment in the literature, it is variously conceptualized as a process or outcome, an end state or a means to an end, a capacity [32,33,34,35,36], a matter of gaining power, and as a matter of agency. Given the broad scope of concerns surrounding empowerment, scholars have examined many topics across a wide range of contexts [33]. Studies have examined many topics, including educational attainment [37], political participation [38], gender-based domestic violence [39], resource control [40,41,42], entrepreneurialism [43], well-being [44], household decision-making [45,46,47], time poverty [48], and health [49,50]. While women’s empowerment is intrinsically important, studies in developing countries have shown that empowering women can also improve children’s health and education [51], decrease child mortality [52], improve the organizational effectiveness of businesses [53], increase agricultural productivity [54], and increase economic growth and reduce poverty [55]. The empowerment of women and poverty reduction are intertwined under the framework of empowerment. A growing amount of evidence has shown that empowerment is instrumental toward reducing income and consumption poverty [34]. Empowering woman as a member of a poor household, in turn, contribute to empower the whole household and thus the issue is a vigorous researchable area in a developing country such as Bangladesh.

## 3. Research Design

### 3.1. The Survey Area, Selection of Participants, and Data Collection

The quantitative research method was used for the present study. Survey data were collected from the northern part of Bangladesh, as the poverty rate was reported the highest in this part of the country [56]. In the first stage, four divisions out of nine divisions of Bangladesh were selected purposively, i.e., Rajshahi, Rangpur, Mymensingh, and Sylhet divisions. Then, from each division, three districts were randomly selected. After that from each district, one Upazila was selected. In the next stage, two villages were randomly selected from each Upazila from the complete list of villages as recorded by Upazila authorities. Finally, from each village, forty households were selected based on the random sampling technique where women were the respondent for the study. The aggregate sample was 960 women. However, some observations were excluded from the analysis, owing to missing data. For this reason, the analysis comprised 914 samples for which complete information was available. Face-to-face interviews of respondents were conducted from May to August 2019 with the help of a peer review and a well-structured interview schedule. We restricted our investigation to married women only as conventional indicators and measures of empowerment characteristically focus on the circumstances of marriage [30], and empowerment within marriage is resulting in the economic wellbeing and health safety of women and their families [57].

### 3.2. Indicators of Women’s Empowerment

Indicators of women’s empowerment were identified based on extensive literature reviews (see Table S1) as follows: household decision-making power, gender attitude, and beliefs, physical mobility, control over resources, and relative freedom from domination by the family.

#### 3.2.1. Household Decision-Making Power

To assess women’s decision-making power within a household, a set of questions were asked relating to (1) large household purchases; (2) purchases for daily needs; (3) visits to family, friends, and relatives; (4) decisions on spending husband’s earnings, and (5) decision-making on their own health care. For each question, if women by themselves or jointly (together with their husband) participate in the decision-making process, the responses were coded as one. If women do not participate in the decision-making process, the responses were coded as zero.

#### 3.2.2. Gender Attitude and Beliefs

Women were asked whether or not a husband is justified to physically abuse his wife under the following circumstances: (1) wife goes out without her husband’s permission; (2) wife argues with her husband; (3) wife refuses to have sexual relations with her husband; (4) the food is burnt when the wife is cooking, and (5) wife neglects the children. For each question, the responses were reverse-coded. If women responded negatively, it was scored one; and otherwise, zero.

#### 3.2.3. Physical Mobility

The physical mobility of respondents was derived by asking the respondents about their going out alone to the following five places: (1) market; (2) friends’ and relatives’ houses; (3) children’s school; (4) health care center or hospital; and (5) outside of the village. If a respondent answered positively, her response was coded as one; and otherwise, as zero.

#### 3.2.4. Control over Resources

Control over resources by women was measured by questioning them about the following conditions: (1) ownership of land and house; (2) ownership of assets; (3) decision-making on the sale or purchase of land, house, and assets; (4) having access to loans, microcredit, and insurance; and (5) being engaged in paid work. If a respondent answered positively, her response was coded as one; and otherwise, as zero.

#### 3.2.5. Relative Freedom from Domination by the Family

To assess relative freedom from domination by the family, the following questions were asked relating to (1) money is taken from them against their will; (2) land or jewelry or livestock is taken from them against their will, and (3) they are prevented from working outside their home. If the women responded that none of the above situations has happened in their lives, they scored one; and otherwise, zero.

### 3.3. Measuring the Poverty Status of Households

This part of the study focused on the poverty level of surveyed women and their households with the help of (1) the classical income poverty approach; and (2) the multidimensional poverty index (MPI). The MPI contains only non-monetary indicators of the three dimensions of poverty, i.e., education, health, and living standards.

#### 3.3.1. Measuring Income Poverty

We examined the impact of women’s empowerment on the income poverty of women and their households, anchored in the poverty line approach, using Foster, Greer, and Thorbecke’s poverty indicators [58]. We converted per capita income in Bangladesh currency (Taka) into US dollars using purchasing power parity. In 2019, the purchasing power parity exchange rate was 1US dollar = 79.12 Taka [56]. If the household’s income was positioned above the poverty line of USD1.90 per day, income poverty was scored zero, and otherwise, one. We evaluated the income poverty gap using the following Equation (1):

(1) $$xi=\frac{p-vi}{p}$$

The poverty line is denoted as *p* in the above equation, and the per capita income of household *i* is denoted as *vi*. Households with income above the poverty line are automatically assigned a value of zero. The income poverty gap is a continuous value, ranging from zero to one [59].

#### 3.3.2. Measuring Multidimensional Poverty

To investigate the households’ deprivation in the poverty dimensions and their ability to meet basic needs, the MPI approach was employed. In association with the United Nations Development Programme, Alkire and Santos [60] developed MPI approach [61]. Among different procedures of multidimensional poverty measurement, namely cluster analysis, factor analysis, weighting procedure, and ordinal approaches [60,62,63], this study used the weighting procedure proposed by Alkire and Santos [60]. The weighting procedure of multidimensional poverty identifies a household’s level of deprivation and the people who face poverty in a community. Finally, the MPI provides absolute poverty levels (in intensity terms) that allow measuring poverty across different settings.

Detailed information about all the included indicators of the poverty dimensions and weighted deprivation as illustrated by Alkire and Santos [60] are presented in Table 1. The MPI includes 10 indicators in three dimensions, which are connected to global standards [64]. For the education dimension, we included two indicators, i.e., school attendance of school-aged children and completion of five years of schooling of all household members. For the health dimension, we used nutrition and child mortality as indicators. Nutrition included the Body Mass Index (ratio of weight in kilograms and square of height in meters) for adults and weight-for-age for children. If any child under the age of five had died in a household, it was treated as a child mortality indicator. The standard of living was measured by focusing on electricity, sanitation facilities, drinking water sources, floor type, cooking fuel, and asset ownership [60].

The weighting procedure of MPI normally requires an equal-weighted dimension and assures an equal-weighted indicator across different dimensions. The poverty cut-off confines the poor by selecting a lower level of deprivation cut-off of 33.33% (1/3). This poverty cut-off reveals a minimum value of weighted indicators to capture multidimensional poor whose deprivation score is equal to or greater than 33.33% [60].

In this study, we calculated different MPI measures for each sample household using one and zero values for each of the 10 indicators. First, we measured the “total deprivation score of each household” by the summation of weighted values for each of the 10 indicators, using weights ranging from zero to one (presented in Table 1). Second, we constructed a “multidimensional poverty dummy,” taking a value of one of the total deprivation score of a household is equal to or greater than a definite threshold of 0.33, and zero otherwise [60]. Finally, we created “multidimensional poverty intensity”, equal to the deprivation score if the sample household is multidimensionally poor (MPI dummy equals 1), and zero, otherwise. The interpretation of the multidimensional poverty intensity is similar to the poverty gap [59].

### 3.4. Estimation Strategy

Data was assembled, coded, tabulated, and analyzed using the STATA 13.1 statistical package to justify the objectives of the study. Descriptive statistics included mean, percentage distribution, and standard deviation for MPI and empowerment indicators. To estimate the impact of women’s empowerment on income poverty and multidimensional poverty, we used the following regression Equation (2):

*y_i_* = *ß*_0_ + *ß*_1_*E_i_* + *ß*_2_*X_i_* + *^e^_i_* (2)

In the above equation, *y_i_* is the income and poverty status for the respondent’s household *i*, *E_i_* is the total empowerment score, *X_i_* is the control variables, and *^e^_i_* is the random error term. We evaluated separate models for each poverty indicator by controlling relevant household and socioeconomic characteristics that may influence poverty. Ordinary least squares (OLS) estimators were used for continuous dependent variables, including income, poverty gap, and multidimensional poverty intensity. Logit estimators were used for binary dependent variables, including income poverty dummy and MPI dummy.

The key coefficient of interest is *ß_1_*, which captures the effect of women’s empowerment on household income and poverty. We expected a positive coefficient or income-increasing empowerment effect when we used household income as the dependent variable and a negative coefficient or poverty-reducing empowerment effect when using poverty indicators. For these analyses, we utilized the SVY command of STATA 13.1 to adjust the village effects.

## 4. Results

### 4.1. Socio-Demographic Analysis

A total of 914 married women participated in this study. The dominant (45.3%) age group was 39–46 years of age, followed by the age group of 29–38 years (38.9%) and 18–28 years (15.8%) (Table 2). As for education level, the women and their husbands were categorized into six groups: (a) illiterate (6.3 and 0.1%, respectively); (b) primary (28.0 and 3.8%, respectively); (c) secondary (37.7 and 7.2%, respectively); (d) higher secondary (13.7 and 32.7%, respectively); (e) graduate (10.6 and 42.7%, respectively); and (f) postgraduate (3.6 and 13.5%, respectively). The average education level was relatively higher for husbands than for women. The majority of the respondents are engaged in paid work (52.4%). On average, four members live in a household and only one member earns money (Table 2).

### 4.2. Evidence of Women’s Empowerment

In this study, we used descriptive statistics to express the respondents’ empowerment status, which also provides an outline of the empowerment variables that are shown in Table 3. In terms of household decision-making, women exhibited impressive evidence of empowerment in their abilities to take decisions solely or jointly with their husband to visit people (80%), to purchase large household products (62%) and daily needs (77%), to spend their husband’s earnings (75%), and their own health care (43%). When a wife disagrees with her husband, physical abuse occurs at a rate of 5%, according to gender attitude and beliefs. Our respondents are less likely to justify physical abuse if women go out without their husbands’ permission (1%), burn food (2%), neglect children (3%), and refuse sex (4%). Our findings are also consistent with a previous study [65], indicating that intimate partner violence has begun to decline in Bangladesh. In terms of freedom of physical mobility, the respondents exhibited substantial empowerment in their abilities to visit hospitals alone (96%), visit relatives (70%), go outside of the village (66%), visit their children’s school (64%), and go to the market (59%). Concerning control over resources, 88% of women have assets, either solely or jointly with their husband and/or others, whereas 28% have access to loans, micro-credit, and insurance facilities. With regards to freedom from family domination, 68% of the respondents do not have permission to work outside.

### 4.3. Evidence of Multidimensional Poverty

Our results show that the majority of households are non-deprived in terms of MPI indicators, such as years of schooling, child’s school attendance, child mortality, nutrition, sanitation, drinking water, and floor, with the exception of cooking fuel facilities. Deprivation scores of households based on the multidimensional poverty index are presented in Table 4.

Surprisingly, no household is deprived of electricity and asset ownership. In line with our findings, DHS [66] reported that 91% of households have access to electricity, and nearly every household owns a single set of assets. Concerning education, 21% of households are deprived of both years of schooling and a child’s school attendance. In terms of the health dimension, only 5% of households are deprived of child mortality indicators. However, the nutrition indicator is quite high (29%). For living conditions, results show that less than 5% of households are deprived in terms of drinking water. There are various sources of drinking water, such as tube well water, pump water, public taps, and so on, and most of the participants are aware of the importance of drinking clean water. Most respondents are accustomed to hygienic sanitation as a result of increased awareness through newspaper reading, media exposure, and promotion of worthwhile and low maintenance cost of hygienic sanitation by government departments. For cooking fuel indicators, more than half of all households (60%) are considered deprived. Because of the availability of local resources, agricultural residue, and animal excreta in Bangladesh, most rural people use these resources for fuel, which makes many rural households to be deprived of cooking fuel indicators.

### 4.4. Impact Assessment on Poverty

Table 5 represents the effects of women’s empowerment on the per capita income of households and estimates of log-transformation of per capita income using the OLS model.

Results are shown as coefficients and their 90% confidence intervals for the variables. Women’s empowerment score has a significantly synergistic (positive) effect on household income. Table 5 reports that a 0.1 increase (10 percentage points) in women’s empowerment would raise per capita income by 14%. Moreover, women’s education, husband’s education, and access to healthcare facilities have significant synergistic effects on both per capita income and log of per capita income. However, preventing women from going outside to work and the number of dependent people in the household has significantly antagonistic (negative) effects on both per capita income and log of per capita income.

Table 6 represents that women’s empowerment score, women’s education, husband’s education, and decision on children’s health and education, all have significantly antagonistic effects on both income poverty and income poverty gap; while experiencing gender violence has a significant synergistic effect on both income poverty and the income poverty gap.

Table 7 represents that empowerment score, women’s education, husbands’ education, women’s asset ownership, engaged in paid work, decision on children’s health and education, and access to healthcare facilities, have significantly antagonistic effects on both multidimensional poverty and multidimensional poverty intensity. However, experiencing gender violence and child mortality have significantly synergistic effects on both multidimensional poverty and multidimensional poverty intensity.

## 5. Discussion

This study has examined the impact of women’s empowerment on poverty reduction in the rural areas of Bangladesh. Different indicators that were used to measure the empowerment status of women are given in Table 3. Our findings show that a 10% increase in women’s empowerment scores will increases per capita income by 14%, while also lowering the prevalence of income poverty by 0.99% and multidimensional poverty by 1.01%, as shown in Table 5, Table 6 and Table 7. It is also found that the education of women and their husbands has a significantly positive relationship with the increase of per capita income and decrease of poverty. The level of education improves people’s participation in the capital and labor markets and is widely recognized as an authentic tool for eliminating poverty and improving the well-being of people [67]. Moreover, qualified women employ themselves in different kinds of prestigious jobs [68], hence reducing their economic dependence and household poverty [69,70]. Generally, highly educated women have more economic and decision-making power and also social freedom, which ultimately reduce their physical, sexual, and emotional vulnerability within the household [71]. Highly educated women can make their own healthcare decisions, ensure better health status for their children, spend their husband’s earnings well, and reject views of physical abuse [72,73]. In addition to this, highly educated household members know a better way to tackle the effect of poverty and chronic poverty [74,75]. The education of women and their husbands acts as a significant catalyst to reduce income poverty, and households with educated couples have a lower risk of being poor [76]. On the other hand, households with a higher number of dependents are multidimensionally poorer than households with a lower number of dependents [60]. Overall, our analysis demonstrates that women and their husbands’ education have a significant effect on reducing income poverty and multidimensional poverty in households. Similar to our study, Sell and Minot [77] stated that education empowers women to hold a better position within the household and to reduce their household poverty.

The measure of household resources (such as the value of a household’s assets) is a long-run measure of economic status rather than a measure of income [78]. Assets, such as mobile phones, televisions, refrigerators, fans, air conditioners, motorcycles, etc., could improve an individual’s health, peace of mind, and mental development, as well as make life more comfortable after a day’s work. Women’s having business equipment (like sewing and embroidery machines, electric irons, incubators, etc.) and skills could improve their income and living conditions as well as decrease household poverty [18]. In line with this result, our study also found that with the increase of assets in the household, multidimensional poverty declines by 0.809%, as shown in Table 7. Our findings support DHS [66], who reported that asset ownership has increased dramatically in both urban and rural areas of Bangladesh, with nearly every household owning a number of assets.

Education is found to be a very important determinant of women’s favorable attitude towards gender equality. The majority of educated women in rural areas work as teachers, are engaged in trade or moneylending, do small businesses, or do sewing and embroidery. We can observe from Table 6 and Table 7 that women engaged in paid work, have a negative correlation with income poverty and multidimensional poverty. Khatun and Kabir [79] studied women’s empowerment in Bangladesh through entrepreneurship and found that women who are not permitted to go outside for work but earn income through running a business such as tailoring, making cakes, gardening, etc. Furthermore, rural women earn income by rearing livestock, money saved from their husband’s family, money received from their natal family, or microcredit from various non-government organizations (NGOs) ultimately increase their economic empowerment [68]. These kinds of income-generating activities empower women not only within the household but also to contribute to reducing income poverty and multidimensional poverty [80].

Intimate partner violence is a crime encompassing physical, sexual, psychological, and emotional abuse by a husband or partner [81]. Frequent abuse is the main cause of deterioration in mental health and happiness [82]. Gender-based violence violates women’s fundamental rights, laws, and regulations, and also limits the potential sources of women’s empowerment. The environment in which women are victims of violence can weaken women’s empowerment [5]. The experience of poverty exacerbates the risk of violence for women [83,84]. However, when men rely on their wives’ financial contributions and maintain a certain standard of living, violence against women decreases [68]. Economic advancements in rural areas have reduced poverty, and households with more assets have a lower rate of domestic violence [78].

Women’s economic independence is an important factor for improving their empowerment. Women have gained freedom of mobility, decision-making power, awareness of their rights as women, and self-confidence from earning and managing their own income [85]. Women in Bangladesh do household work that is not paid work, like working on their husband’s farm and caring for their children. This makes them financially dependent on their husbands and other male relatives [86]. However, it has been found that women’s income significantly increases overall household income and the income of the husband and wife is pooled as family income [17]. Empowered women contribute not only by providing their personal income, but also by assisting their husbands with various income-generating activities, such as working in farms and shops, giving advice, taking loans from various NGOs, and supervising family resources [68]. World Vision Ghana states that if women have the opportunity to get credit for employment, their contribution to the well-being of their family would help to reduce family poverty and women’s economic dependence on their husbands [80]. As a result, when restrictions are imposed on women from working outside the home, the overall income is significantly reduced. Our study has similar findings that prohibiting women from working outside significantly reduces per capita income, as shown in Table 5.

Women’s capability to participate in and make household decisions is one of the key elements of empowerment. Women have the power to make decisions on household expenses (groceries, clothes, and expenditure on children’s health and school fees) in Indonesia, Myanmar, the Philippines, and Thailand, while men occasionally participate in major expenditure decisions [17]. Moreover, credit decisions are also made by mutual agreement of women and men in all the mentioned countries [17]. The results of Wouterse [9] show that when more power is given to women over household expenditure, income spent on food quality increases as opposed to income spent on unhealthy food, recreation, and alcohol. In this study, we find that women’s participation in household decision-making and control over resources, considerably increases per capita income, while at the same time, significantly decreases income poverty and multidimensional poverty, as presented in Table 5, Table 6 and Table 7. Similar findings have been reported by previous studies that women play a significant role in household food security, children’s education, and the healthy lives of household members [17,68]. However, contradictory trends are observed in poor Ghanaian communities, where men have very strong domination over women within the household [80].

In this study, we also observe a significant relationship between child mortality and income poverty and multidimensional poverty in the rural areas of Bangladesh. Women’s relative unequal access to education, employment, finance, decision-making power, basic health care facilities, and other productive resources, is considered as the prime reason for their ill-health and that of their children. Relatively economically poor and less empowered women are less likely to receive proper health care facilities than wealthy and empowered women [87]. According to Lachaud [88], in rural areas, lower living standards of households, in terms of assets, are associated with high child mortality. Furthermore, it has been discovered that living below or slightly above the national poverty line, as well as a lack of insurance coverage, are risk factors for children’s health.

A wealth of research has reported that access to loans and micro-credit has played a remarkable role in reducing poverty [80,89,90]. However, in this study, we find no significant relationship between women’s access to loans and microcredit and per capita income or reducing income poverty, which can be verified from Table 5 and Table 6. Freedom of mobility indicator determines not only the extent to which women can go outside the home but also their personal autonomy in terms of not having to seek permission from their husbands or any other household member [91]. In Bangladesh, women who do not have any freedom to go outside on their own, are possibly deprived of empowerment programs, such as microfinance, and therefore, microfinance oftentimes does not reach the poor [92]. This is a common scenario in rural areas compared to urban areas because, in rural areas of Bangladesh, the purdah practice confines women to the home and compound; they mostly have to obtain permission either from their husband or any other responsible person or at least, tell them when they are going outside. Mahmud et al. [93] reported that women who work outside the home do not usually ask for permission when they go outside for work, but do so when they go out for other purposes.

The different selected indicators considerably influence women’s empowerment status, showing an increment in per capita income and a decrease in different poverty indices. Educated women have more opportunities to participate in income-generating activities, household decision-making, and resource control, which help to empower them. Empowered women are more conscious of their own rights as well as of the wellbeing of their children and family members, which eventually can reduce household income poverty and multidimensional poverty. If women become conscious about their rights and supportive policies that empower women socioeconomically are in place, that would generate a righteous succession of women’s overall empowerment in both their household and societal spheres.

## 6. Conclusions

Women’s empowerment is a critical human rights issue with implications for the well-being of women, their families, and society as well as socio-economic development and poverty reduction, especially in developing countries like Bangladesh. In this paper, we have examined the impact of women’s empowerment on income, income poverty, and multidimensional poverty in the rural area of Bangladesh. Data were collected from 914 respondents. To measure women’s empowerment, this study used different indicators which were selected based on an extensive literature review. The results reveal that women’s empowerment score contributes to increase per capita income and decrease income poverty and multidimensional poverty. Women’s education significantly reduces multidimensional poverty and income poverty. Whereas gender violence, taking resources against women’s will and preventing women from working outside, caused a considerable decline in per capita income and, increase in income poverty and multidimensional poverty. Working women who earn cash have high household autonomy, high freedom of movement, no gender preference and they face lower levels of domestic violence. Violence against women remains one of the most widespread and persistent human rights abuses in the world, stemming from deep-rooted notions of women’s unequal status. It is a major obstacle to the fulfillment of women’s and girls’ human rights and the achievement of the 2030 Agenda for Sustainable Development. Violence against women is no longer viewed as an inevitable part of family life, of social relations, of the workplace, or war; indeed, violence against women cannot be justified under any circumstances. However, the disempowerment of women in Bangladesh is not solely linked to gender, because working women who earn cash have high household autonomy, high freedom of movement, no gender preference and they do not rationalize domestic violence, all of which indicate that women’s empowerment is positively associated with lower levels of domestic violence.

The concept of empowerment is closely related to agency and thereby to human development. Any policy aiming at human development needs to be informed about the factors that enhance agency and contribute to empowerment as both agency and empowerment are not only intrinsically valuable but also instrumentally important for poverty reduction. The government and non-government organizations’ programs directed to repositioning family planning services should be geared up and centered around various dimensions of women’s empowerment, especially focusing on their economic existence in society and decision-making power. Educational programs should prioritize achieving gender equity in schooling outcomes. Although this study examines a number of indicators accompanied by women’s empowerment, such as asset ownership, employment status, household decision-making power, educational status, physical mobility, and so on. Future research could add other different indicators, like assets brought to the marriage, proximity to other family members, characteristics of the respondent’s parents and upbringing, and the influence of community norms, which could explain empowered women’s roles in household poverty reduction. The findings may be useful as a policy tool for planners, administrators, and development workers to initiate appropriate strategies for women’s empowerment to reduce poverty in the rural areas of Bangladesh, which can also be applied in other similar contexts.

## Figures and Tables

**Table 1:** Dimensions, indicators, deprivation cut-off, and weights of the multidimensional poverty index.

Dimension	Indicator	Description and Deprivation Cut-Off	Relative Weight
Education	Years of schooling	No household member has completed five years of schooling	1/6
	Child’s school attendance	Any school-aged child is not attending school in years 1 to 8	1/6
Health	Mortality	Any child who has died in the family	1/6
	Nutrition	Any adult to child for whom there is nutritional information is malnourished *	1/6
Living standard	Electricity	The household has no electricity	1/18
	Sanitation	The household’s sanitation facility is not improved (according to MDG guidelines), or it is improved but shared with another household **	1/18
	Drinking water	The household does not have access to safe drinking water (according to MDG guidelines) or safe drinking water is more than a 30-min walk from home (round-trip) ***	1/18
	Floor	The household has dirt, sand, or dung floor	1/18
	Cooking fuel	The household cooks with dung, wood, or carbon	1/18
	Asset ownership	The household does not own one of the following assets: radio, TV, telephone, bicycle, motorbike, refrigerator, and does not own a car or truck.	1/18

* Adults are considered malnourished if their BMI is below 18.5. Children are considered malnourished if their z-score of weight-for-age is below minus two standard deviations from the median of the reference population. This was estimated following the algorithm provided by the WHO Child Growth Standards (WHO, 2006). http://www.who.int/childgrowth/software/en/ (accessed on 27 January 2020). ** A household is considered to have access to improved sanitation if it has some type of flush toilet or latrine, or ventilated improved pit or composting toilet, provided that they are not shared. *** A household has access to safe drinking water if the water source is any of the following types: piped water, public tap, borehole or pump, protected well, protected spring or rainwater, and it is within a distance of a 30-min walk (round-trip).

**Table 2:** Socio-demographic analysis.

Variables		Percentage	Mean	Standard Deviation
Age	18–28	15.8	2.295	0.724
	29–38	38.9		
	39–46	45.3		
Education	Illiterate	6.3	3.05	1.191
	Primary	28		
	Secondary	37.7		
	Higher secondary	13.7		
	Graduate	10.6		
	Postgraduate	3.6		
Husband’s education	Illiterate	0.1	4.54	0.952
	Primary	3.8		
	Secondary	7.2		
	Higher secondary	32.7		
	Graduate	42.7		
	Postgraduate	13.5		
Employment status	Engaged in paid work	52.4	0.524	0.499
	Unpaid work	47.6		
Household size		914	3.906	0.859
Earning member		914	1.41	0.492

**Table 3:** Descriptive statistics of women’s empowerment.

Indicators of Women’s Empowerment	Yes (%)	Mean	Standard Deviation
**Women’s participation in household decision-making**			
Making decisions on large household purchases	62.0	0.623	0.485
Making decisions on purchases for daily needs	77.0	0.774	0.419
Making decisions on visits to family, relatives, or friends	80.0	0.799	0.401
Making decisions on spending husband’s earnings	75.0	0.753	0.432
Making decisions on own health care	43.0	0.434	0.496
**Attitude towards wife-beating**	8.0	0.923	0.266
Wife beating is justified if she goes out without telling her husband	1.0	0.987	0.114
Wife beating is justified if she argues with husband	5.0	0.950	0.219
Wife beating is justified if she refuses to have sex with her husband	4.0	0.964	0.187
Wife beating is justified if she neglects the children	3.0	0.967	0.178
Wife beating is justified if she burns the food	2.0	0.983	0.131
**Physical mobility**			
Going alone to the market	59.0	0.591	0.492
Going alone to visit friends, family, and relatives	70.0	0.697	0.460
Going alone to visit a health care center or hospital	96.0	0.956	0.204
Going alone outside of the village	66.0	0.656	0.475
Going alone to visit children school	64.0	0.635	0.482
**Control over resources**			
Ownership of land	53.0	0.526	0.500
Ownership of assets	88.0	0.875	0.331
Decision on sale and purchase of house, land, and assets	57.0	0.567	0.496
Have access to loans, micro-credit, and insurance	28.0	0.277	0.448
Engaged in paid work	52.0	0.524	0.500
**Relative freedom from domination by the family**			
Money and jewelry taken against her will	2.0	0.984	0.127
Land taken against her will	2.0	0.976	0.153
Prevented from working outside the home	68.0	0.323	0.468

Notes: Answer "Yes" means positive outcome for indicators of women’s participation in making household decisions, indicators of physical mobility, and indicators of control of resources; while answer "Yes" means negative outcome for indicators of attitude towards wife-beating and relative freedom from domination by the family.

**Table 4:** Descriptive statistics of MPI elements.

MPI Indicator	Deprived (%)	Non-Deprived (%)	Mean	Standard Deviation
Years of schooling	21.0	79.0	0.208	0.406
Child school attendance	22.0	78.0	0.218	0.413
Mortality	5.0	95.0	0.046	0.209
Nutrition	30.0	70.0	0.299	0.458
Electricity	0.0	100.0	0.000	0.000
Sanitation	10.0	90.0	0.103	0.304
Drinking water	4.0	96.0	0.036	0.187
Floor	34.0	66.0	0.342	0.475
Cooking fuel	59.0	41.0	0.589	0.492
Asset ownership	0.0	100.0	0.000	0.000

**Table 5:** Effect of women’s empowerment on per capita income.

Variables	Per Capita Income	Log of per Capita Income
	OLS	OLS
Empowerment score	1.483 * (0.786)	0.008 ** (0.003)
Education of respondents (years)	5.840 *** (2.011)	0.032 *** (0.009)
Education of husband (years)	6.046 *** (2.245)	0.038 *** (0.010)
Asset ownership of women (dummy)	2.321 (6.809)	0.002 (0.029)
Engaged in paid work (dummy)	−0.493 (3.920)	0.002 (0.017)
Experiencing gender violence (dummy)	−13.330 (15.099)	−0.121 * (0.065)
Political knowledge (dummy)	10.759 (10.415)	0.058 (0.045)
Control over use of household income (dummy)	12.168 (7.740)	0.058 * (0.033)
Prevented from working outside (dummy)	−12.303 ** (4.797)	−0.044 ** (0.021)
Money and jewelry taken against their will (dummy)	−14.761 (14.024)	−0.044 (0.060)
Access to loans, micro-credit, and insurance (dummy)	1.105 (4.692)	−0.002 (0.020)
Decision on children’s health and education (dummy)	2.612 (4.338)	0.039 ** (0.019)
Access to healthcare facilities (dummy)	5.948 *** (2.169)	0.020 ** (0.009)
Child mortality (dummy)	2.559 (8.270)	−0.014 (0.035)
Number of dependent persons in the household (number)	−7.764 * (4.565)	−0.033 * (0.020)
Constant	9.912 (19.247)	1.422 *** (0.082)
(Pseudo) R-squared	0.090	0.151
Observations	914	914

Notes: Coefficient estimates are reported. Standard errors are shown in parentheses. * Significant at 10% level; ** Significant at 5% level; *** Significant at 1% level.

**Table 6:** Effect of women’s empowerment on income poverty.

Variables	Income Poverty (Dummy)	Income Poverty Gap (0–1)
	Logit	OLS
Empowerment score	−0.099 *** (0.037)	−0.005 ** (0.002)
Education of respondents (years)	−0.465 *** (0.107)	−0.015 *** (0.005)
Education of husband (years)	−0.617 *** (0.111)	−0.027 *** (0.006)
Asset ownership of women (dummy)	−0.169 (0.301)	0.014 (0.018)
Engaged in paid work (dummy)	−0.342 * (0.195)	−0.016 (0.010)
Experiencing gender violence (dummy)	1.791 ** (0.858)	0.105 *** (0.040)
Political knowledge (dummy)	−0.344 (0.591)	−0.023 (0.027)
Control over use of household income (dummy)	0.022 (0.331)	−0.041** (0.020)
Prevented from working outside (dummy)	−0.031 (0.243)	0.007 (0.013)
Money and jewelry taken against their will (dummy)	0.146 (0.624)	−0.011 (0.037)
Access to loans, micro-credit, and insurance (dummy)	0.050 (0.233)	0.005 (0.012)
Decision on children’s health and education (dummy)	−0.818 *** (0.205)	−0.054 *** (0.011)
Access to healthcare facilities (dummy)	−0.246 ** (0.104)	−0.002 (0.006)
Child mortality (dummy)	0.609 (0.383)	0.043 ** (0.022)
Number of dependent persons in household (number)	0.506 ** (0.228)	0.020 (0.012)
Constant	5.402 *** (0.910)	0.377 *** (0.051)
(Pseudo) R-squared	0.212	0.188
Observations	914	914

Notes: Coefficient estimates are reported. Standard errors are shown in parentheses. * Significant at 10% level; ** Significant at 5% level; *** Significant at 1% level.

**Table 7:** Effect of women’s empowerment on multidimensional poverty.

Variables	Multidimensional Poverty (Dummy)	Multidimensional Poverty Intensity (0–1)
	Logit	OLS
Empowerment score	−0.101 ** (0.046)	−0.567 ** (0.224)
Education of respondents (years)	−0.306 ** (0.124)	−2.019 *** (0.574)
Education of husband (years)	−0.481 *** (0.133)	−2.136 *** (0.640)
Asset ownership of women (dummy)	−0.809 ** (0.365)	−6.764 *** (1.943)
Engaged in paid work (dummy)	−0.543 ** (0.244)	−2.148 * (1.119)
Experiencing gender violence (dummy)	2.511 *** (0.818)	15.683 *** (4.309)
Political knowledge (dummy)	−0.861 (0.866)	−2.339 (2.972)
Control over use of household income (dummy)	−0.295 (0.404)	0.166 (2.209)
Prevented from working outside (dummy)	−0.382 (0.321)	0.267 (1.369)
Money and jewelry taken against their will (dummy)	0.822 (0.713)	7.634 * (4.003)
Access to loans, micro-credit, and insurance (dummy)	0.323 (0.294)	1.499 (1.339)
Decision on children’s health and education (dummy)	−2.703 *** (0.240)	−21.482 ** (1.238)
Access to healthcare facilities (dummy)	−0.376 *** (0.128)	−1.591 ** (0.619)
Child mortality (dummy)	2.709 *** (0.418)	23.409 *** (2.360)
Number of dependent persons in the household (number)	0.270 (0.298)	1.329 (1.303)
Constant	5.516 *** (1.089)	51.766 *** (5.493)
(Pseudo) R-squared	0.344	0.426
Observations	914	914

Notes: Coefficient estimates are reported. Standard errors are shown in parentheses. * Significant at 10% level; ** Significant at 5% level; *** Significant at 1% level.

## Data Availability

The data that support the findings of the study are available from the corresponding author upon reasonable request.

## Supplementary Materials

The following are available online at https://www.mdpi.com/article/10.3390/ijerph18136909/s1, Table S1: Indicators of women’s empowerment in previous studies.
