# Supplementary material for: The Influence of Women’s Empowerment on Poverty Reduction in the Rural Areas of Bangladesh: Focus on Health, Education and Living Standard
Source: Int J Environ Res Public Health. 2021 Jun 27;18(13):6909. doi: 10.3390/ijerph18136909 (PMC8293807; doi:10.3390/ijerph18136909)
Supplement: Supplementary file 1 [file ijerph-18-06909-s001.zip › ijerph-1247292-supplementary.pdf]

Table S1. Indicators of women's empowerment in previous studies.

Indicators of women's empowerment	Previous studies
Women's participation in household decisions making	
Making decision on large household purchases	Habibov et al. (2017); Hashemi et al. (1996); Miedema et al. (2018); Schuler et al. (2010).
Making decision on purchases for daily needs	Habibov et al. (2017); Hashemi et al. (1996); Miedema et al. (2018); Schuler et al. (2010).
Making decision on visits to family, relatives, or friends	Gupta and Yesudian (2006); Habibov et al. (2017); Mahmud et al. (2012); Miedema et al. (2018).
Making decision on husband earnings spending	Habibov et al. (2017); Miedema et al. (2018).
Making decision on own health care	Gupta and Yesudian (2006); Habibov et al. (2017); Miedema et al. (2018).
Attitudes towards wife beating	
Wife beating justified if she goes out without telling her husband	Gupta and Yesudian (2006); Habibov et al. (2017); Mahmud et al. (2012); Miedema et al. (2018).
Wife beating justified if she argues with him	Habibov et al. (2017); Mahmud et al. (2012); Miedema et al. (2018).
Wife beating justified if she refuses to have sex with him	Habibov et al. (2017); Miedema et al. (2018).
Wife beating justified if she neglects the children	Gupta and Yesudian (2006); Habibov et al. (2017); Mahmud et al. (2012); Miedema et al. (2018).
Wife beating justified if she burns the food	Gupta and Yesudian (2006); Habibov et al. (2017); Mahmud et al. (2012); Miedema et al. (2018).
Physical mobility	
Going alone to visit market	Ganle et al. (2015); Hashemi et al. (1996); Mahmud et al. (2012); Schuler et al. (2010).
Going alone to visit friends, family & relative	Hashemi et al. (1996); Mahmud et al. (2012); Schuler et al. (2010).
Going alone to visit health care centre or hospital	Ganle et al. (2015); Hashemi et al. (1996); Mahmud et al. (2012); Schuler et al. (2010).
Going alone to visit outside of village	Ganle et al. (2015); Hashemi et al. (1996); Schuler et al. (2010).
Going alone to visit children school	Patrikar et al. (2014).
Control over resources	
Ownership of land	Hashemi et al. (1996); Schuler et al. (2010).
Ownership of asset	Hashemi et al. (1996); Malapit and Quisumbing (2015); O'Hara and Clement (2018); Schuler et al. (2010); Sraboni and Quisumbing (2018).
Decision on sale & purchase of house, land and asset	Malapit and Quisumbing (2015); O'Hara and Clement (2018); Sraboni and Quisumbing (2018).
Have access to loan, micro-credit and insurance	Mahmud et al. (2012); Malapit and Quisumbing (2015); O'Hara and Clement (2018); Sraboni and Quisumbing (2018).
Engaged in paid work	Ganle et al. (2015); Mahmud et al. (2012); Miedema et al. (2018).
Relative freedom from domination by the family	
Money & jewellery taken against her will	Ganle et al. (2015); Hashemi et al. (1996); Schuler et al. (2010).
Land taken against her will	Hashemi et al. (1996); Schuler et al. (2010).
Prevented from working outside the home	Hashemi et al. (1996); Schuler et al. (2010).

Advantages of weighting procedure of multidimensional poverty index.

The weighting procedure of multidimensional poverty spots the household's level of deprivation and the people who face poverty in a community. For some following advantages, we follow this procedure: First, a multidimensional poverty measure is vigorous for both ordinal variables and cardinal variables with the classification of deprived and non-deprived individuals. Second, it assures dimensional monotonicity [12] to adjust the intensity with the incidence of multidimensional deprivation of poverty. Third, it facilitates poverty contrasts across mutually exclusive and exhaustive population subgroups. Fourth, the censored and uncensored headcount ratios can be crashed by the indicator of deprivations to assist to understand overall poverty structures. Finally, this measure circulates the reflection of the weighted deprivation ratio of poverty that the poor people experience [13].

References

1. Habibov, N.; Barrett, B.J.; Chernyak, E. Understanding women's empowerment and its determinants in post-communist countries: Results of Azerbaijan national survey. *Womens. Stud. Int. Forum* **2017**, *62*, 125–135, doi:10.1016/j.wsif.2017.05.002.
2. Hashemi, S.M.; Schuler, S.R.; Riley, A.P. Rural Credit Programs and Women'S Empowerment in Bangladesh. *World Dev.* **1996**, *24*, 635–653, doi:10.1016/0305-750X(95)00159-A.
3. Miedema, S.S.; Haardörfer, R.; Girard, A.W.; Yount, K.M. Women's empowerment in East Africa: Development of a cross-country comparable measure. *World Dev.* **2018**, *110*, 453–464, doi:10.1016/j.worlddev.2018.05.031.
4. Schuler, S.R.; Islam, F.; Rottach, E. Women's empowerment revisited: A case study from Bangladesh. *Dev. Pract.* **2010**, *20*, 840–854, doi:10.1080/09614524.2010.508108.
5. Gupta, K.; Yesudian, P.P. Evidence of women's empowerment in India: A study of socio-spatial disparities. *GeoJournal* **2006**, *65*, 365–380, doi:10.1007/s10708-006-7556-z.
6. Mahmud, S.; Shah, N.M.; Becker, S. Measurement of Women's Empowerment in Rural Bangladesh. *World Dev.* **2012**, *40*, 610–619, doi:10.1016/j.worlddev.2011.08.003.
7. Ganle, J.K.; Afriyie, K.; Segbefia, A.Y. Microcredit: Empowerment and disempowerment of rural women in Ghana. *World Dev.* **2015**, *66*, 335–345, doi:10.1016/j.worlddev.2014.08.027.
8. Patrikar, S.R.; Basannar, D.R.; Seema Sharma, M. Women empowerment and use of contraception. *Med. J. Armed Forces India* **2014**, *70*, 253–256, doi:10.1016/j.mjafi.2013.12.014.
9. Malapit, H.J.L.; Quisumbing, A.R. What dimensions of women's empowerment in agriculture matter for nutrition in Ghana? *Food Policy* **2015**, *52*, 54–63, doi:10.1016/j.foodpol.2015.02.003.
10. O'Hara, C.; Clement, F. Power as agency: A critical reflection on the measurement of women's empowerment in the development sector. *World Dev.* **2018**, *106*, 111–123, doi:10.1016/j.worlddev.2018.02.002.
11. Sraboni, E.; Quisumbing, A. Women's empowerment in agriculture and dietary quality across the life course: Evidence from Bangladesh. *Food Policy* **2018**, *81*, 21–36, doi:10.1016/j.foodpol.2018.09.001.
12. Alkire, S.; Foster, J. Counting and multidimensional poverty measurement. *J. Public Econ.* **2011**, *95*, 476–487, doi:10.1016/j.jpubeco.2010.11.006.
13. Alkire, S.; Santos, M.E. Measuring Acute Poverty in the Developing World: Robustness and Scope of the Multidimensional Poverty Index. *World Dev.* **2014**, *59*, 251–274, doi:10.1016/j.worlddev.2014.01.026.
